# iDNA from terrestrial haematophagous leeches as a wildlife surveying and monitoring tool – prospects, pitfalls and avenues to be developed

**DOI:** 10.1186/s12983-015-0115-z

**Published:** 2015-10-01

**Authors:** Ida Bærholm Schnell, Rahel Sollmann, Sébastien Calvignac-Spencer, Mark E. Siddall, Douglas W. Yu, Andreas Wilting, M. Thomas. P. Gilbert

**Affiliations:** Centre for GeoGenetics, Natural History Museum of Denmark, University of Copenhagen, Copenhagen, Denmark; Center for Zoo and Wild Animal Health, Copenhagen Zoo, Frederiksberg, Denmark; Leibniz Institute for Zoo and Wildlife Research, Berlin, Germany; Department of Forestry and Environmental Resources, North Carolina State University, North Carolina, Raleigh USA; Epidemiology of highly pathogenic microorganisms, Robert Koch Institute, Berlin, Germany; Sackler Institute of Comparative Genomics and Division of Invertebrate Zoology, American Museum of Natural History, New York, USA; School of Biological Sciences, University of East Anglia, Norwich Research Park, Norwich, UK; State Key Laboratory of Genetic Resources and Evolution, Kunming Institute of Zoology, Chinese Academy of Sciences, Kunming, Yunnan China; Trace and Environmental DNA Laboratory, Department of Environment and Agriculture, Curtin University, Perth, Western Australia Australia; Present address: US Forest Service, Pacific Southwest 17 Research Station, 1731 Research Park Drive, Davis, CA 95618 USA

## Abstract

Invertebrate-derived DNA (iDNA) from terrestrial haematophagous leeches has recently been proposed as a powerful non-invasive tool with which to detect vertebrate species and thus to survey their populations. However, to date little attention has been given to whether and how this, or indeed any other iDNA-derived data, can be combined with state-of-the-art analytical tools to estimate wildlife abundances, population dynamics and distributions. In this review, we discuss the challenges that face the application of existing analytical methods such as site-occupancy and spatial capture-recapture (SCR) models to terrestrial leech iDNA, in particular, possible violations of key assumptions arising from factors intrinsic to invertebrate parasite biology. Specifically, we review the advantages and disadvantages of terrestrial leeches as a source of iDNA and summarize the utility of leeches for presence, occupancy, and spatial capture-recapture models. The main source of uncertainty that attends species detections derived from leech gut contents is attributable to uncertainty about the spatio-temporal sampling frame, since leeches retain host-blood for months and can move after feeding. Subsequently, we briefly address how the analytical challenges associated with leeches may apply to other sources of iDNA. Our review highlights that despite the considerable potential of leech (and indeed any) iDNA as a new survey tool, further pilot studies are needed to assess how analytical methods can overcome or not the potential biases and assumption violations of the new field of iDNA. Specifically we argue that studies to compare iDNA sampling with standard survey methods such as camera trapping, and those to improve our knowledge on leech (and other invertebrate parasite) physiology, taxonomy, and ecology will be of immense future value.

## Introduction

Gathering knowledge on the abundance, dynamics and distributions of species is one of the fundamental challenges for conservation biologists who aim to apply and assess the impacts of management interventions. At the root of this challenge lies the need to monitor species over space and time. Currently, a number of different survey and monitoring methods are being applied to terrestrial vertebrates, ranging from physical immobilization with possible invasive sampling (e.g. capture for telemetry, biopsies, etc.) [1] to non-invasive and often indirect sampling (e.g. scat, hair, sound or sign surveys and camera-trapping) [2–5]. Non-invasive methods are often preferred for ethical and practical reasons.

In recent years, interest has grown in the application of environmental DNA (eDNA) as a non-invasive tool with which to obtain biodiversity information (e.g. [6–10]). eDNA refers to DNA that can be extracted from environmental samples (e.g., air, water, soil) without needing the target organisms themselves. When subjected to metabarcoding, identification of multiple species from a single bulk sample is possible [11]. Within the general discipline of eDNA lies iDNA, “invertebrate-derived DNA”, where vertebrate genetic material is extracted from invertebrates. The invertebrates that have been used in iDNA studies are diverse, including mosquitoes [12, 13], carrion and blow flies [14, 15], midges [16], ticks [17], and terrestrial leeches [18]. Similarly diverse are their habitats, behaviours and diets, which range from flesh-eating and haematophagous (blood-sucking) to coprophagous (feces-eating) and saprophagous (eating dead/decaying organic matter) [19]. Preliminary studies have demonstrated that iDNA can recover information about vertebrates across a broad range of taxa with different sizes and ecologies, with detection sensitivity largely representing only a single meal per leech (I.B. Schnell unpublished data and [14, 19]), indicating iDNA has potential to survey and monitor vertebrates.

**Fig. 1:**
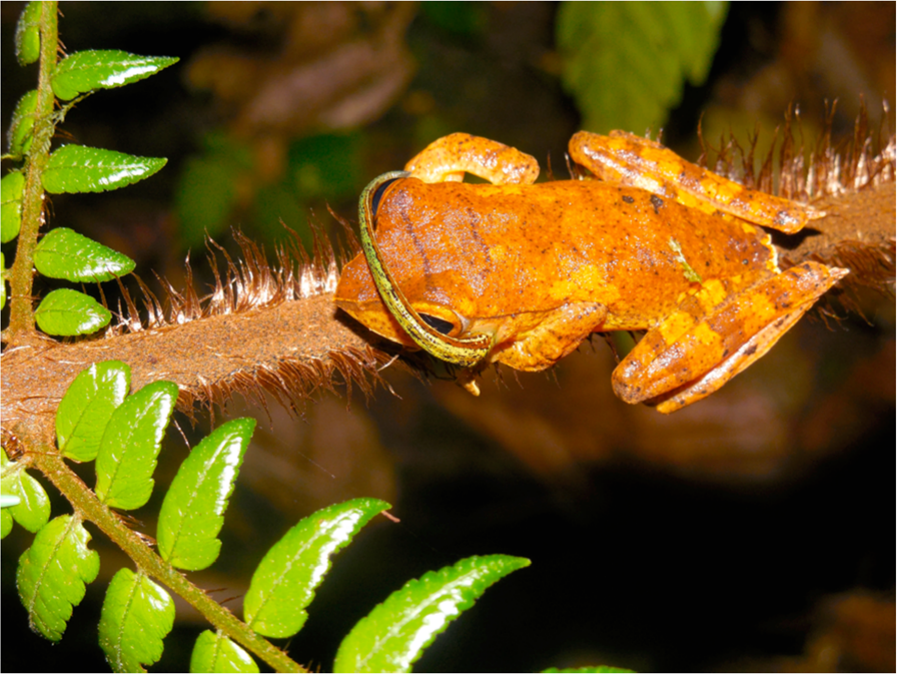
A terrestrial haematophagous leech (*Haemadipsa* spp.) sampling vertebrate biodiversity (*Rhacophorus* spp.). Courtesy Andrew Tilker (IZW).

Among the potential sources of iDNA, haematophagous terrestrial leeches (Fig. 1) have received considerable recent interest from the conservation biology sector. Relevant species belong to the family Haemadipsidae, within the suborder Hirudiniformes [20–22], and members of this family occupy large parts of Asia, Australasia, and Madagascar [20, 23] - areas that are known for their extensive tropical rainforests, rich biodiversity and number of endemic or threatened vertebrate species [24–26]. Given that monitoring efforts are often severely hampered by the limited economic support available, one of the most attractive benefits of using leech-derived iDNA as a tool is that collection is cheap, rapid, and requires no special skills or equipment, allowing easy recruitment of personnel from local people. The leech collector simply offers his/herself up for bait. Valuable equipment is not at risk of being damaged or stolen - unlike camera traps - and there is no need for batteries or CO_2_ - unlike mosquito traps. Furthermore, in addition to their ease of sampling, their generally high abundance allows collection of large sample sizes. In tropical forests during the wet season, it is not unusual for single collectors to collect hundreds of leeches per day. Given that such leeches feed on vertebrate taxa spanning a broad range of sizes and ecologies (I.B. Schnell unpublished data), bulk processing of leeches through simply digesting the entire leech, purifying the total DNA then subjecting it to metabarcoding PCRs [18] combined with high-throughput sequencing, provides an efficient means to assess vertebrate species richness. The number of leeches collected over any survey area is also a direct measure of sampling effort, making it easy to quantify and compare effort across sites and visits. Lastly, relatively high detection success of vertebrate iDNA has been reported for terrestrial leeches. In contrast to invertebrates with high metabolism and short inter-meal intervals such as flies and mosquitoes, terrestrial leeches (and ticks) only feed a few times annually and to some extent possess the ability to retard the rate of DNA degradation [18, 27]. Preliminary observations indicate very high detection rates of well-preserved DNA in leeches [18], suggesting that identification of sex or discrimination between individuals may be possible.

The steep increase of camera-trapping studies since the 1990s, when camera-traps were first used systematically to study wildlife, highlights the demand for reliable, non-invasive vertebrate survey techniques. The advantages of haematophagous terrestrial leeches as a source of iDNA raises the hope that they could complement, or in some instances even outperform camera-trapping. As such, they could become an important component of studying tropical rainforest biodiversity, for example in the context of evaluating the biodiversity co-benefits of sustainable forest certifications such as FSC and carbon storage payments such as REDD+.

Despite these apparent benefits and the burgeoning interest in their application, and although the recovery of DNA derived from invertebrate blood meals has been used in the biomedical context for a number of years - for example to reveal whether various haematophagous invertebrates act as vectors of infectious diseases [27] – the application of iDNA in general, and leech iDNA specifically to vertebrate species surveying and monitoring remains relatively undeveloped. In particular, how iDNA data might be combined with state-of-the-art analytical tools such as site-occupancy and spatial capture-recapture (SCR) models to estimate wildlife abundance, population dynamics and distributions has not been addressed. The frameworks of capture-recapture (CR) and occupancy modelling were developed to address our imperfect ability to count the individuals in a population (CR; e.g. [28, 29]) and to detect a species’ presence at a sampling location in the first place (occupancy; e.g. [30, 31]). However, the correct use of these modelling frameworks relies on key assumptions being met by the data. These assumptions centre on temporal and geographic closure (referring to “static-ness” of the measure of interest, for example abundance), the independence of records, and, usually, the absence of false-positive detections. Thus, although the potential power of iDNA has led to considerable excitement in the applied ecology and conservation community, the roles that the innate biology of the invertebrates (in particular their behaviour and life-cycle) and the biases in the data generation methods (including PCR amplification, sequencing, and data processing) play with regards to whether iDNA data can be analysed using the existing methods, remain unexplored.

In this review, we discuss the challenges that face the application of existing analytical methods/models to iDNA, in particular possible violations of key assumptions. Although the issues discussed relate to all sources of iDNA, we focus on the concrete example of terrestrial haematophagous leeches to illustrate the advantages and disadvantages of iDNA. We then review the implications of leech biology for occupancy and capture-recapture models, and outline developments that are needed to determine if leech iDNA can meet the assumptions of those methods. Finally, we address how the analytical challenges associated with leeches may apply to other sources of iDNA.

### Leech-introduced sampling biases

No survey tool is equally suitable to study all species, as every tool has its inherent sampling biases and uncertainties. Camera traps, for example, are well suited for surveying medium to large sized terrestrial animals. Nevertheless, where and how camera traps are set up influences which species are more likely to be photographed [32]. In the context of using leeches to inform modelling approaches, a key question therefore is which sampling biases would be introduced as a result of their inherent biology. To date, very few studies have focused on the digestive biology of terrestrial leech species belonging to Haemadipsidae. This introduces uncertainties that have to be considered during both leech collection and subsequent data analysis. Uncertainties include those related to whether leeches exhibit feeding biases as a result of (i) host-specificity and habitat preference, and (ii) the discrepancy between time and location of leech collection versus time and location of feeding (the discrepancy arising due to movement of the leech during the time that has passed since its last feed until it is collected).

#### Host-specificity and habitat preferences

**Fig. 2:**
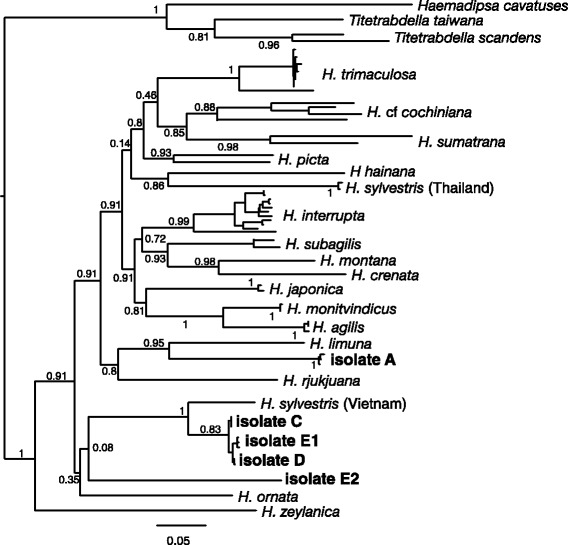
Mitochondrial tDNA-based phylogenetic tree including sequences from five leeches reported in [18]. These leeches were collected at a single location in the Annamite Mountains, Vietnam and apparently belong to at least 3 distinct genetic clades

Terrestrial haematophagous leeches have been shown to feed on a wide range of vertebrate species, including birds, amphibians, reptiles and mammals [23, 33]. Although a degree of host-specificity has been recorded for some terrestrial leech species – for example, the species *Tritetrabdella taiwana* seems to feed primarily on amphibians [34] – it is still not fully understood whether this is a true host preference, or simply an outcome of what animals are present in the same (micro-) habitats as the leeches. Behavioural studies have demonstrated that while coexisting Bornean brown (*Haemadipsa sumatrana*) and tiger leeches (*Haemadipsa picta*) both feed on mammals (with no apparent preferences shown within mammals), brown leeches live on the ground, and tiger leeches usually sit on leaves of small trees and bushes [34, 35]. Consequently, tiger leeches are less likely than brown leeches to feed on small, fossorial terrestrial mammals. Thus, even if terrestrial haematophagous leeches are opportunistic feeders, any given species likely will not feed on all vertebrates in an area evenly, and the general lack of knowledge about leech taxonomy (Table 1 and Fig. 2), phylogeography, and behaviour makes it challenging to account for interspecific ecological differences among leeches in both collection and subsequent data analysis.

**Table 1:** A brief introduction to the challenge of leech taxonomy

The relative phylogenetic relationships of the genus *Haemadipsa* that result from maximum likelihood analysis of combined cox1 and cox3 mitochondrial data (Fig. 2) reveal a variety of challenges associated with the use of iDNA. The proper identification of species in the family Haemadipsidae relies both on internal sexual characteristics [72, 76, 77] and on external patterns in color, annulation and eyespot arrangements [20, 73, 74]. Consequently, field collection with rapid assessment are limited to the designation of morphospecies; aggregations of specimens that look, but are not necessarily genetically, alike. Species assignments and delimitations on the basis of DNA barcodes necessarily are predicated on the accuracy and precision of the information available in public databases [75, 80]. Both of these desiderata are undermined by incomplete coverage across taxonomic groups of interest, by the persistence of misidentified isolates in public databases, by the existence of undiscovered diversity and by cryptic species.

#### Temporal and spatial discrepancies between feeding and collection

Camera traps provide time-stamped records of which vertebrate species were present at known spatial locations - haematophagous terrestrial leeches do not. Because of long inter-meal intervals and possible leech movement after feeding, it is impossible to determine exactly how far away the parasitised host (i.e., the target species) is from the location of leech collection. The time since last feeding event is not fixed but can be influenced by leech species, size of last blood meal, how many previous feedings the leech has had, and even leech size and age [23, 36].

Climate is another factor influencing inter-meal intervals. Temperature, humidity and light intensity affect leech species in different ways, but a common observation is that leeches living in aseasonal environments have a more uniform feeding behaviour compared to leech species living in seasonal environments [23]. Although terrestrial haematophagous leeches are believed to be mostly quiescent between feeding events, limited movement to more or less moist areas has been observed (M. Siddall unpublished data), and passive movement on a host during a failed feeding attempt is also plausible. Whether these are significant sources of error depends on how large the leech movements are relative to the movement of the target species, and the spatial resolution of the study. For example, it might not be possible to study microhabitat associations of target species that exhibit little movement themselves, but macro-habitat associations of wide-ranging species should not be impacted by small-scale leech movements. In summary, while leech iDNA clearly offers the potential to provide a fast and relatively cheap overview of vertebrate species present in an area, its power as a survey tool will benefit from increased understanding of the taxonomy, distribution, and behaviour of terrestrial haematophagous leech species.

### Biases associated with the generation and identification of sequence data

Challenges to iDNA studies (regardless of the source) at the molecular analytical level predominantly relate to the occurrence of false positives and false negatives. These errors can occur both during sequence data generation and in subsequent processing.

#### Sequence-based data generation

Avoiding contamination of single leech samples with other sources of DNA represents the principal challenge during iDNA sequence generation. Contamination may be derived from contact with the handler, or within the laboratory in which they are analysed, as both could result in the generation of false-positive detections. Contamination principally arises due to the fact that, although leech iDNA is generally of good quality, it is more degraded and present at much lower concentration than DNA from fresh tissues, and particularly in analyses relying on PCR amplification coupled with second generation sequencing, some level of (cross)-contamination with other sources of DNA, especially amplicons, seems unavoidable. However, laboratory based contamination can be limited/identified if (i) material is analysed following ancient or forensic DNA guidelines, (ii) by personnel skilled in the analysis of degraded materials, (iii) incorporating replicate analyses, negative DNA extraction and PCR controls, and (iv) introducing a degree of conservatism during bioinformatic analyses [37]. A useful measure is the adoption of ‘blocking’ probes to prevent for example human DNA amplifying during PCR [38], since its presence in a sample provides no useful ecological information, given that collectors are likely to add their DNA to samples.

#### Sequence data analyses

No matter what iDNA information is available from leeches, its potential use is dependent on the sensitivity and specificity of the methods used to target the iDNA, and subsequently, the quality of reference databases against which the iDNA sequences are compared. Currently, species-diagnostic reference DNA sequences exist for only a fraction of the vertebrate species living in the tropical rainforests and other ecosystems inhabited by terrestrial leeches. While this problem can be resolved in part simply by generating more barcode markers from more species, the deeper issue is that even for relatively well-studied groups of mammals, some studies indicate that the number of identified species is highly underestimated in comparison to the true diversity [39], and as long as species are not recognized as such, the quantity of missing DNA sequences in the public DNA reference databases cannot be assessed. Therefore, as with other eDNA and iDNA studies, continued efforts to both refine species taxonomy and documentation, as well as genetic characterization of those missing from public DNA databases is essential.

Furthermore, many sequences published in Genbank are unreliable [40, 41]. Many contain PCR or sequencing errors and un-excised primer sequences or cloning vectors. Furthermore, the taxonomic assignment given to numerous databased sequences is inaccurate, either due to initial misidentification of the source specimen, contamination of samples during DNA sequence generation, or incorrect labelling of nuclear mitochondrial insertions as true mitochondrial sequences (or vice versa). Finally, information about the geographic origin of the source specimen is often absent from the database, thus hampering phylogeographic assignment of sequences to populations, subspecies, or even species (in cases where species have been split after submission of the sequence). Thus, appropriate curating of both newly generated and pre-existing DNA sequences in the public databases must take a key role within the rapidly expanding number of barcoding initiatives.

Challenges at the genetic analysis stage can limit many assignments to higher taxonomic ranks at best, or result in false positive and false negative species detections. In particular, false positives significantly bias statistical analyses [42], while lack of high taxonomic resolution prevents species-level inference. Many ecosystems, especially tropical forests, harbour multiple species of the same genus/subfamily, with one of the species often being much rarer and/or threatened. For example in the Central Annamites in Vietnam, the endangered large-antlered muntjac *Muntiacus vuquangensis* occurs sympatrically with the more common northern red muntjac *Muntiacus vaginalis*. If these species are combined into the same operational taxonomic units (OTUs), iDNA data cannot directly inform the conservation of the threatened species.

#### Individual identification

Non-invasive genetic individual-level studies have been applied to a diverse array of animal taxa, using DNA isolated from a range of sample types including hair (e.g. [43, 44]), feathers (e.g. [45]), and faeces (e.g. [46, 47]). Several authors have discussed the pitfalls of using individual identification based on genetic markers in capture-recapture models and the effects of possible misidentification on abundance estimates of target species. Among others, these challenges include (i) allelic dropout, in which one of the two alleles of a heterozygous individual fails to amplify (false identification), and the (ii) shadow effect, where the genotype from one individual is indistinguishable from that of a previously captured animal (e.g. [48, 49]). Whereas to date most studies have used microsatellite loci, recent technical developments have increased the number of single nucleotide polymorphism (SNP) loci that can be studied per unit cost. It is likely that the higher number of SNP loci can reduce the pitfalls associated with earlier microsatellite-based individual identifications. The generally well preserved DNA in terrestrial haematophagous leeches ensures that both such marker types can be amplified from at least a proportion of samples (I.B. Schnell unpublished data), thus potentially enabling identification of individuals. However, identification based on leech iDNA might be even more challenging than for most other non-invasive DNA sources, which usually originate from only one individual. More than one individual of the same vertebrate species might be present when analysing the leech iDNA, either within the same leech, or within the same pool of leeches if multiple leeches are analysed as a single unit. Although the first is unlikely for a rare species, the latter seems more likely - often pools of leeches must be analysed as single units so as to reduce analysis costs to a reasonable level. If two individuals from a single species are co-amplified, the resulting mixed profiles will represent artificial genetic diversity, a similar problem to the allelic dropout. This in turn will lead to overestimation of the species’ population size.

### From random leech collection to organized surveys

Given our current knowledge about both the certainties and uncertainties of leeches as a sampling tool, and the limitations associated with how the iDNA can be translated into vertebrate data, a fundamental question is how (or even whether) leech iDNA can be used in combination with existing analytical tools, given the idiosyncrasies of sampling invertebrates and the assumptions and requirements of these tools. To address this, one must first consider what analytical tools are available to survey species and populations. At the simplest level, one might ask whether a particular target vertebrate species is present in an area of interest. The most basic approach to answering this question is through direct identification of the target species – something for which there now exists a number of well developed techniques including single-species approaches such as PCR coupled to cloning/Sanger sequencing, or qPCR using species-specific probes, or metabarcoding approaches that are based around deep amplicon sequencing using High Throughput Sequencing Platforms [11]. With such data, one can subsequently consider those species detected as present, and those not detected as absent, although such approaches suffer from both leech specific, and more general weaknesses. From the leech-specific angle, the above discussed challenges relating to generalist feeding preferences [23, 33], render it hard to collect leeches in a manner that would enable efficient targeting of specifically chosen vertebrate species. The obvious general weakness is that failure to detect a vertebrate species is not proof of its absence (e.g. [31]). A variety of models have been developed that take into account the imperfect detection probabilities, so as to overcome such “false absences”, both at the species (occupancy) and the individual levels (capture-recapture).

Ultimately, the exact analytical tool to use depends on the goal of a particular study, and the data to be collected depends on the analytical tool of choice [50, 51]. We therefore discuss leech iDNA in the context of occupancy and spatial capture-recapture models, because they (a) account for imperfect detection, (b) are widely used in the field of wildlife monitoring, and (c) present fields of on-going model development. Both approaches are widely used on data derived from camera trapping and other (mostly) non-invasive survey approaches, and, as discussed below, require specific consideration before they can be applied to leech iDNA. See Table 2 for an overview of the basic assumptions in the analytical tools described in this paper.

**Table 2:** Common analytical tools and their basic assumptions

	Presence	Occupancy modelling	Spatial capture-recapture
Goal/Measure	Is a particular species present?	Probability of occurrence/distribution; multi-species occupancy modelling for species richness	Abundance/density
Important assumptions		Independence of samples (both spatial and temporal); occupancy state at each site remains constant during the study (‘closure assumption’); for multi-species occupancy, sampling of different species must be independent of each other	Population closure; detections are independent among individuals
Data requirements	Stratified sampling of different habitat types to maximize detection probability	At least 20 sites, at least some (random) with repeated visits	Individuals are identifiable; several (at the very least 5) individuals, recaptures at different locations
Key literature		MacKenzie DI, Nichols JD, Hines JE, Knutson MG and Franklin AB [52]	Royle JA, Chandler RB, Sollmann R and Gardner B [62]

#### Presence surveys

We refer to presence surveys as those that are only concerned with establishing either the presence of a single focal species in a larger study area (for example, a national park), or a species count for an area. In both cases, raw data are used without accounting for imperfect species detection (so no inference should be made concerning species *absence*), and there is no interest in determining habitat associations or abundances. In this case, leech collection does not need to follow a specific sampling design. If interest is on a single species, its (assumed) preferred habitat can be targeted; if interest is on a species count, sampling should be stratified to increase the chance of detecting species with different ecological requirements.

The additional complication of iDNA is that detection of a vertebrate species in a leech is not proof of its presence in a specific location *on the day of collection* but instead provides evidence only of its presence in a larger area, the size of which depends on typical vertebrate *and* leech movements within a timeframe. For the leech, the effect of movement depends on feeding intervals and blood retention/DNA preservation times. Considering the very limited and coarse inference that is desired from simple presence surveys, leech movement after feeding and blood retention likely does not present much of a problem.

#### Occupancy models

**Fig. 3:**
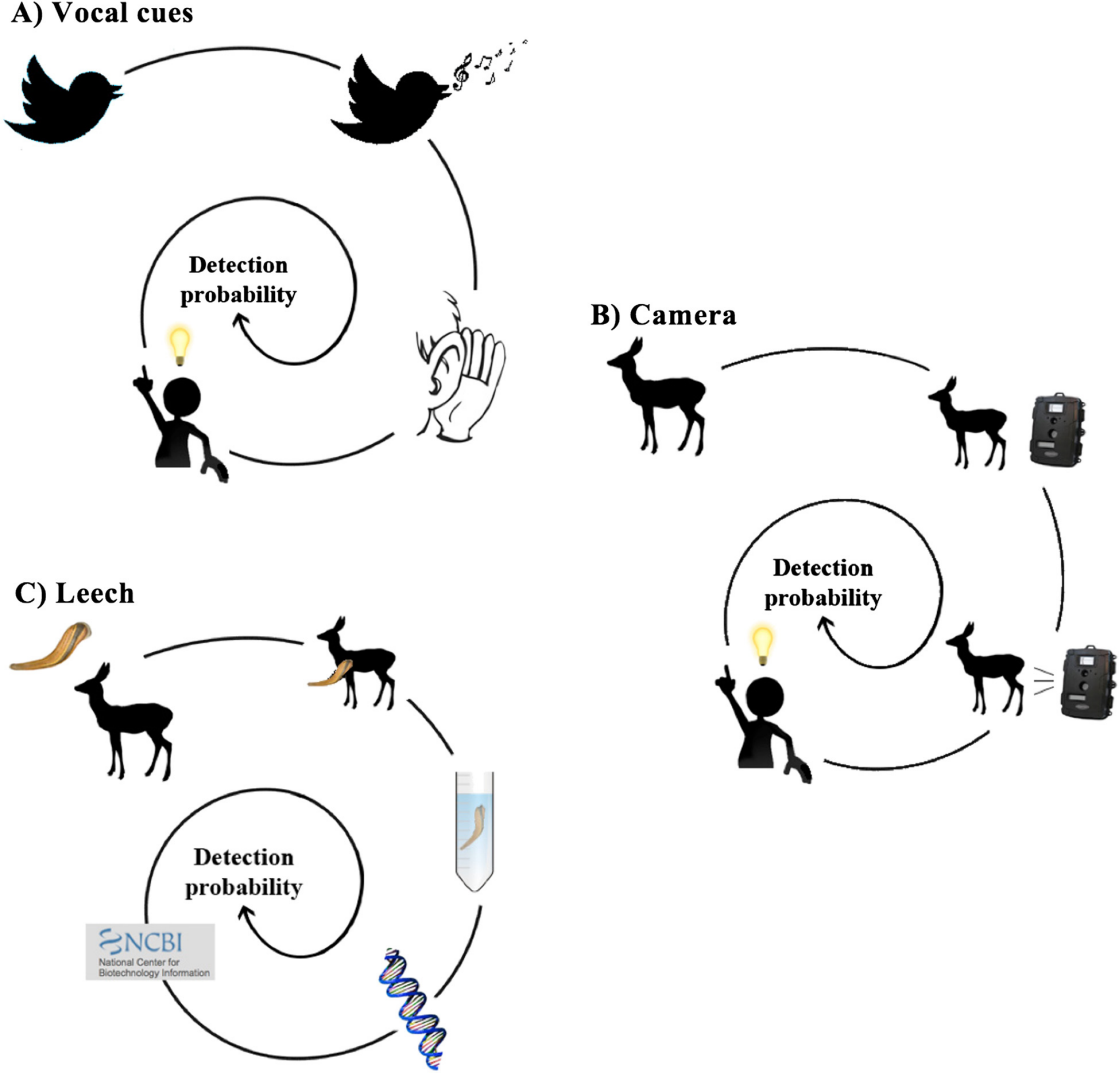
Components influencing detection probability. Panels represent three selected detection methods: **a** Vocal cues, **b** Camera traps and **c** Leech collection assuming presence of target species (*and leech species*). With vocal cues **a**, detection probability constitutes of p (*Focal species sing*), p (*Observer hears song*) and p (*Correct identification of species by the observer*). For camera traps (**b**) it is p (*Focal species goes in front of camera trap*), p (*Camera is being triggered*) and p (*Correct identification of species by the observer*). When using iDNA from leeches (*and most other invertebrates*) **c**, the detection probability constitutes of p (*Leech feed on focal species*), p (*Fed leech collected*), p (*Target DNA extracted/amplified*) and p (*Correct identification based on the DNA*)

Occupancy models [30, 52] treat species observations only as detections versus non-detections, rather than concluding presence versus absence. Over repeated visits to a collection of sampling sites, which are used to compensate for imperfect species detection, species detection/non-detection data can be used to estimate the probability of true species occurrence. Both occupancy and detection probability can furthermore be modelled as functions of environmental covariates (see Table 3 and Fig. 3 for aspects that can influence target species detection probability in leech-based occupancy studies). Occupancy models provide estimates of the percentage of the total area occupied (PAO) and the probability of occupancy at any given site according to environmental covariates, which are parameters of great interest for many wildlife management programs.

**Table 3:** Detection probability in leech-based occupancy modelling

The question of leech habitat use versus target species habitat described in ‘Occupancy modelling’ points towards an important aspect: the definition of detection probability in leech-based occupancy studies. The most basic definition of detection probability in occupancy models is the probability that the species is detected, given that it is present [31]. However, in any realistic situation, the probability structure is much more complex. For example, if birds are detected by song, the probability of detection constitutes two pieces *i)* the probability that a bird sings and *ii)* the probability that the song is detected by an observer and correctly assigned to a species (Fig. 3a-c illustrates the components that contribute to the detection probability for some common survey methods compared to leeches). In the case of leech sampling, the series of probabilities leading to species detection is even more complex (Fig. 3c). On the basic level, *i)* both the leech and target species have to be present at the sampling site. One is not necessarily conditional on the other, but it seems reasonable to suspect some correlation between the two (leeches should not occur where there are no hosts). Leech habitat preferences, for example, feed into the probability of a leech being present at a sampling site. Conditional on both leech and target species being present, *ii)* the leech then has to feed on the target species, and here, possible leech host preferences can influence detection. On the next level, *iii)* a collector has to detect the leech. The probability of detecting a leech could be influenced by habitat, weather, time of day and ability of the collector. Conditional on a leech filled with remnants of blood of the target species being collected in the field, then come *iv)* the lab related probabilities – that DNA can be extracted, amplified and correctly identified to species level. For example, if a reference sequence of a particular species is missing it is impossible to match the sequences obtained from the sample, and thus the species will be not detected. In the analysis of the resulting species detection/non-detection data, all these levels of the detection process get balled up into a single “detection probability”. It is important to keep in mind the different processes that feed into this parameter, as variation in any of these levels across sampling sites or times or even leeches, can lead to biased results if not accounted for. Fortunately, methods such as occupancy modeling enable detection probability to be modeled as a function of spatial and temporal covariates. This is regardless of where in the process (e.g. from biology of the leech to sensitivity of genetic assay) these occur. For example, covariates could include measurable variables (both continuous or categorical) related to the genetic analyses, such as amount of blood extracted from a leech, measures of DNA quality, etc. These measures, however, may not be as straightforward to obtain when pools of leeches constitute a sample, rather than a single leech.

Key assumptions of occupancy models concern the spatial independence of sampling sites and the independence of repeated visits to a particular site. Further, the occupancy state of each site is assumed to be constant over the course of the survey (the ‘closure’ assumption). But note that with data from repeated surveys, dynamic occupancy models can be used to estimate probability of site extinction and colonization [52]. When applied to multiple species, community occupancy models can be used to estimate species richness (e.g. [53, 54]). In this case, the independence of species is an additional assumption.

Under the assumptions that (i) only DNA from the last blood meal is detected and (ii) the movement of leeches after detaching from their last host is negligible, spatial independence becomes predominantly a question of survey design and the spatial ecology of the host species. Leeches should be collected at locations that are spaced far enough apart to avoid spatial autocorrelation induced by the movement of individuals of the target species. The independence of repeated visits to a particular site seems to be well approximated considering that at every visit to a site, a new set of different individual leeches is collected.

The question of constant occupancy is harder to tackle with leeches. Laboratory-based experiments using absolute quantification real-time PCR assays targeting short (ca 150 bp) mitochondrial DNA fragments derived from known species meals have shown that vertebrate DNA can be obtained from medicinal leeches at least 4-5 months after feeding [18]. If these observations hold true with wild terrestrial haematophagous leech species, the time of leech collection and the time of target-species presence could differ by months. Leeches collected at one site likely also have fed at different times in the past, further complicating the question of temporal resolution of the data. Changes in occupancy status of a site (i.e., going from occupied to unoccupied, or vice versa) during the survey violate the assumption of ‘population closure’ and leads to complex biases in estimates of occupancy [55]. Assuming constant occupancy patterns over larger time frames may not be particularly problematic in relatively stable systems of intact tropical evergreen forests, but may pose a bigger problem in disturbed and unstable habitats. The difficulties in defining the temporal frame of the population under study should be kept in mind. Knowledge on feeding rates and intervals, as well as host DNA retention in the field (in essence, sensitivity to detection through the molecular tool used) would help quantify this potential problem.

When the aim of the research is to analyse data of multiple species jointly in a community occupancy analysis, the limited number (possibly only one with certainty) of species that can be detected in a single leech poses a problem. If species A is found in a leech, it is no longer possible to detect any other species in that same leech, and detection of species is therefore no longer independent. This situation also arises with other survey methods, for example box traps, where once an individual/species has been caught, that trap closes and is no longer available for other individuals/species to be captured in. A manner to alleviate this dependency in species detections is to pool multiple leeches per site and visit. Each leech can “capture” one species, and the more leeches that are pooled together at each visit, the more species that can potentially be detected, reducing the amount of dependency among species. However, without additional information on the size and evenness of the sampled community and possible leech feeding preferences, it is impossible to advise on the number of leeches that should be pooled per sample.

Another important assumption of occupancy models is that data do not contain false positives (false negatives, i.e. failure to identify a species from DNA, are readily handled by occupancy models, as long as they occur at random, because they just constitute a different form of not observing the target species). Taxonomic assignment methods should therefore be conservative rather than overly optimistic. Occupancy models allowing for false positives have been developed, but the practical implementation of early models [42] is limited [56, 57], whereas the application of most recent models requires additional information on the false-positive generating process [58, 59].

#### Capture-recapture models

Capture-recapture (CR; e.g. [28, 29]) methods, including spatial capture-recapture (SCR; e.g., [60–63]), make use of individual-level detection data to estimate individual detection probability in order to obtain unbiased estimates of abundance or density. When used on temporally repeated datasets, capture-recapture data can further be used to investigate population dynamics (e.g. [64]). We focus this discussion on the assumptions and data requirements of single-season (i.e., closed population) SCR models, because these constitute an improvement over traditional closed population methods, specifically when it comes to surveying rare and wide ranging species (e.g. [65]). By making use of spatial information associated with individual detections, these models are able to account for variation in individual exposure to the sampling array, as well as animal movement. Key assumptions of SCR models that need careful consideration when using leeches as a sampling tool include independence of detections among individuals and demographic closure of the study population during the study period. In contrast to occupancy models, where each individual should only be detected at one sampling site, SCR models require multiple recaptures of the same individual at several sampling sites to estimate a movement-related parameter that is part of the detection model.

Similar to occupancy surveys, we have to assume that the leech location is representative of the location of the individual target animal detected within the leech. As long as only the last meal is detected (or can be distinguished from previous meals), and leech movement is negligible relative to target species movement, this assumption seems reasonable. Several SCR studies using search-encounter type methods, rather than fixed location detectors, have analysed detection data by discretising space into grid cells and assigning detections to grid cell centre points (e.g., [66]). As long as the resolution of the grid was narrower than animal movement, resolution had little to no effect on density estimates, suggesting that within these limits, SCR models are robust to uncertainties about the location of an individual detection [62].

Independence in detection among individuals is more problematic. A leech that has fed on an individual will likely not feed on another individual for quite some time, thus becoming unavailable to other individuals in the study area. This presents a situation similar to the species-level dependency in detection we described for community occupancy models. In SCR models, a sampling device that can only hold a single individual is termed a single-catch trap, and to date there is no formal model describing the detection process for these kinds of traps. However, failure to account for the dependencies in detection induced by single-catch trap type detectors does not seem to cause bias in estimates of density, particularly when trap saturation (the percentage of full traps in a given sampling occasion) is low [60]. Assuming that leeches are hyper-abundant relative to their hosts, we believe there is little chance for “leech saturation”. Dependence in individual detections can further be decreased by pooling multiple leeches (see also community occupancy models above), thus allowing several individuals to be detected simultaneously. Leeches differ from typical single-catch traps in that individuals can be “captured” (i.e., detected) in more than one leech, and therefore at more than one survey location, in a given sampling occasion. To our knowledge, nobody has attempted to investigate whether these characteristics of leech iDNA collection have any impact on density estimates from SCR models.

Potentially the largest issue with leech collection for SCR analysis is the difficulty of defining a temporal sampling frame, because the ages of detected blood meals are unknown. The individual we detect in a leech may have died or emigrated since it served as a host, thus violating the assumption of population closure. Animals that become unavailable for sampling during the survey (because they die or emigrate), or that become available only well after the survey was initiated (because they get recruited) cause bias in estimates of detection probability, which translates into biased estimates of abundance/density. Without (i) the ability to age blood meals and (ii) knowledge about the feeding intervals of wild terrestrial leeches, it currently seems difficult to work around this problem, and this should be kept in mind as a potential source of bias when using SCR on leech data.

Lastly, leech-based (S) CR methods are subject to possible genetic misidentification error. In non-spatial capture-recapture, efforts have been made to formally deal with genetic misidentification (reasons for genetic misidentification are described in the paragraph “Individual identification”) [67–69]. To our knowledge, genetic misidentification issues have not yet been addressed in an SCR framework.

### General discussion

While a potential wealth of information can be extracted from leeches, ultimately the economics of conservation biology mandate that output information must be viewed in light of gains made. In short, as financial resources spent on monitoring often directly diminish those available for conservation actions [70, 71], investment in leeches as a source of iDNA can only be justified if they are to provide reproducible and reliable information. In this context a number of topics that relate to both information about leeches, and the analytical methods themselves, urgently require addressing so as to ensure this is possible.

#### Future research priorities - Leech taxonomy, ecology, physiology

At perhaps the most basic level lies improvement to current leech species identification and taxonomy (Table 1). This is a complex task that will require resolving conflicting historical names and describing new species. Multiple unrelated leech lineages are called by a single name, and a few species exist that are known by multiple names across their geographic range. In addition there are both likely cryptic leech species, and a host of undescribed species that require attention (e.g. [22, 72–75]).

In addition to taxonomy, an improved understanding of the seasonal occurrences and abundances of leech species, as well as potential habitat and feeding preferences, is important. In this regard, knowledge of leech movement would be invaluable for tackling questions relating to the spatial resolution of target species observations, as required by subsequent analytical methods. Improved information on leech biology and taxonomy (e.g. [20–23, 72–77]), is also key for determining whether ecological groups of leeches can be defined that exhibit similar behaviour with regards to time since last feeding event, movement after feeding event, and potential host or habitat associations. Until proven otherwise, it should be assumed that leeches of different species (and possibly even size and age within a species) behave differently. It should be noted that the existence of ecologically different groups of leeches can be accounted for in subsequent analyses, as long as they are separated from each other (as far as possible based on current knowledge) for genetic processing.

#### Considerations for study design

While leech collection is a non-invasive means to survey their vertebrate hosts, it is of course very invasive for the leeches themselves. Therefore, careful consideration should be given to study design, and to ensuring reliable downstream analyses. In this way, unnecessary collections that could potentially significantly affect leech populations can be avoided. Basic information such as the percentage of collected leeches that contain amplifiable DNA in different environments, altitudes and seasons, as well as differences between leech species, represent important factors to be incorporated into any study design. It is important to note that leech sampling not only provides data on vertebrates, but also yields information that could improve our knowledge of leech ecology (e.g. [23, 75]). Variation in leech detections across space could be incorporated into models of leech abundance and distributions providing insight into habitat associations of leeches.

#### Leeches and occupancy modelling

Despite some potential difficulties, it appears that leech samples sufficiently approximate the basic assumptions of occupancy modelling when used within a well-planned study. The key open question is to what extent leech habitat preferences limit the application of occupancy models. Obviously, it is impossible to use leeches as a tool to study vertebrates in habitats where leeches do not occur. If heterogeneous landscapes are studied, where leech habitat and non-leech habitat are interspersed, inference on occupancy of the non-leech habitat by a vertebrate target species is impossible. But even within leech habitats, it is possible that leeches prefer certain conditions, e.g. moist and shady, and are therefore more likely to be found in one spot than in another [23]. If we think about an individual leech as a sampling device, then pools consisting of fewer leeches translate into a lower probability of detecting the target species in a pool, simply because they represent fewer “sampling devices”. A straightforward way to avoid potential bias stemming from leech habitat preferences is to standardise the number of leeches collected per site and visit, so that target species detection probability is not influenced by the number of leeches collected, although areas with low leech densities will naturally increase logistical costs. Alternatively, the number of leeches found in a sampling plot could be used as a covariate on target species detection probability. A lower probability of encountering a leech at a given site then translates into a lower detection probability of the target species (Table 3). This is analogous to, for example, accounting for the number of days a camera-trap was functional within a sampling occasion when estimating species detection probability in camera-trap based occupancy models.

It is also uncertain as to whether individual leeches collected at a single site during a single visit could be used as ‘repeat visits’ for the purpose of single-species occupancy models. When using single leeches, we expect that the detection probability of a given species would be close to zero, which leads to parameter estimation problems in occupancy models. Leeches, however, have the advantage that even when collected at the same point in space and time, it is still conceivable that they constitute independent samples, because it is unlikely that they have fed on the same animal and dropped off their last host at the same time and place. Therefore, if a large number of leeches can be collected during a single visit, they could be split into subsets, which then constitute the’repeat visits’. This would greatly reduce required time effort in the field, especially in remote areas, as a single visit could provide the necessary repeated observations.

#### Leeches and spatial capture-recapture modelling

There is potential to obtain SCR data from leeches, and this would tremendously increase our ability to study populations of rainforest mammals: Only in a small fraction of species can individuals be visually distinguished on camera-trap photographs; the moist and hot climate renders scat-based genetic collection and identification from some species difficult to impossible; and the vast majority of species is too rare and/or elusive for alternative observation-based methods such as distance sampling. On the other hand, when using methods like camera-trapping, it is possible to effectively target certain groups of animals, such as predators, by placing cameras on well-defined trails (e.g. [78, 79]). Such targeted collection is not possible with leeches. At this time we are not aware of any information from the field giving insight into how likely it is to detect the same individual twice when collecting leeches. Thus, it may turn out that this method is financially prohibitive for SCR modelling, particularly of rare species, because obtaining an adequate sample size would require sequencing a huge number of leeches individually. While continuing decreases in sequencing costs should allow for commensurate increase in leech screening ability, it may be that suitable sample sizes remain out of reach for other reasons (e.g. limited storage capacity, concerns for leech populations).

#### Extrapolation from leeches to other sources of iDNA

As mentioned in the introduction, other sources of iDNA have been studied as well (e.g. [12–17]). Even though several other invertebrates seem to hold promise as a tool for sampling vertebrates, many of the same considerations and potential biases exist if they are to be used as a systematic vertebrate surveying tool. Invertebrates with high metabolism such as flies and mosquitoes have shorter inter-meal intervals than leeches or ticks, resulting in higher temporal resolution of target species detections. Flying, on the other hand, likely results in larger movements of the invertebrates, increasing uncertainty about the actual location of the target species. Vertebrate DNA derived from saprophagous insects further pose the problem that the detected target animal is dead and thus its “occurrence” is not directly shown [19]. This problem potentially also applies to coprophagous invertebrates, which, if feeding on carnivore faeces, may yield DNA of consumed prey [14]. A short overview of the most important traits of four different invertebrates in relation to suitability as a source of iDNA-based species or individual observation data is provided in Tables 4 and 5.

**Table 4:** Terrestrial haematophagous leeches versus other sources of iDNA [13, 15, 16, 81–89]

	Terrestrial leeches	Blow/Flesh flies	Mosquitos	Ticks
Geographical distribution	The Indo-Pacific, incl. Madagascar, Australia and Tasmania	Worldwide	Worldwide	Worldwide
Invertebrate activity	Seasonal variation (moisture dependent)	Seasonal variation	Seasonal variation	Seasonal variation
Collection efficiency	High	High	Medium	Low
Collection method	By hand/trap	Traps	Traps	By hand/nets
Diet	Haematophagous	Saprophagous Coprophagous Flesh-eating	Haematophagous	Haematophagous
Potential feeding bias	Between classes of vertebrates, species dependent	Between classes of vertebrates, primarily mammals	Between and within classes of vertebrates, single host specificity documented, species dependent	Between and within classes of vertebrates, single host specificity documented, species dependent
Size of “host”-meal	<15 mL	<60 μL	2-10 μL	<1 mL
Multiple detectable meals	Possibly	Yes	Yes	Yes
Percentage of samples containing amplifiable host DNA	20–85 %	20–50 %	40–80 %	40–50 %
Host individual ID	Yes	Yes (1 %)	Yes (30 %)	N/A
Temporal scale (Time last meal remains detectable)	Months–1 year	Days	<1 week	Weeks - 10 months (depending on life cycle stage)
Spatial scale (Distance from last “meal” to collection site)	< 1 km	< few kilometres	Few meters	Few meters

**Table 5:** How do different iDNA sources fit into the discussed analytical tools and their basic assumptions?

	Terrestrial leeches	Blow/Flesh flies	Mosquitos	Ticks
Presence	Suited, but possibly not ideal for arboreal species	“Suited” (risk of identifying carcasses)	Suited but restricted to preferred hosts, if any	Suited but restricted to preferred hosts, if any
Occupancy	Suited but potential violation of constant occupancy assumption	Collection location not necessarily equal to host location (risk of identifying carcasses)	Collection location not necessarily equal to host location	Suited but potential violation of ‘closure’ assumption
(S) CR	Potential violation of population closure assumption	Collection location not necessarily equal to host location; very low success rate of microsatellite-based genotyping (risk of identifying carcasses)	Collection location not necessarily equal to host location	Suited (?)

## Conclusions

Terrestrial haematophagous leeches hold considerable potential as a tool upon which to base vertebrate surveys and monitoring programs. Nevertheless, before their potential can be fully exploited, considerable effort is required to improve our understanding of leech taxonomy, biology and behaviour. With this information secured, the fundamental assumptions upon which the state-of-the-art analytical methods rest can be re-assessed, and decisions can be made about the true potential and limits of the leech system. Naturally the challenges facing leech iDNA are not unique to leeches, and in many cases can be directly extrapolated to other invertebrate sources of iDNA (Tables 4 and 5). Until these challenges are resolved, it will, however, be critical to continue pilot studies - only application in the field will reveal the amount of data (species and individual level detections) that can realistically be collected using appropriate and logistically feasibly sampling protocols (sufficient sampling sites and repeat visits within a reasonable time frame). In particular, we advocate the need for *in situ* comparisons of leech (or indeed, any) iDNA tools with other standard survey methods, including camera trapping, other sources of eDNA and human surveys. With this information in hand, researchers will be able to assess the relative strengths and weaknesses of iDNA versus such methods, and in doing so apply combinations of existing tools so as to maximize the data generated in biodiversity assessment and monitoring studies.

## Abbreviations

CR: Capture-recapture
eDNA: Environmental DNA
iDNA: Invertebrate-derived DNA
SCR: Spatial capture-recapture
